# Neuroinflammation and the Female Brain: Sex-Specific Mechanisms Underlying Mood Disorders and Stress Vulnerability

**DOI:** 10.3390/life16010139

**Published:** 2026-01-15

**Authors:** Giuseppe Marano, Claudia d’Abate, Gianandrea Traversi, Osvaldo Mazza, Eleonora Gaetani, Rosanna Esposito, Francesco Pavese, Ida Paris, Marianna Mazza

**Affiliations:** 1 Unit of Psychiatry, Fondazione Policlinico Universitario A. Gemelli IRCCS, 00168 Rome, Italy; 2 Department of Neurosciences, Università Cattolica del Sacro Cuore, 00168 Rome, Italy; 3 Department of Molecular and Developmental Medicine, Obstetrics and Gynecological Clinic, University of Siena, 53100 Siena, Italy; 4 Unit of Medical Genetics, Department of Laboratory Medicine, Ospedale Isola Tiberina-Gemelli Isola, 00186 Rome, Italy; gianandrea.traversi@gmail.com; 5 Spine Surgery Department, Bambino Gesù Children’s Hospital IRCCS, 00168 Rome, Italy; osvaldo.mazza1973@hotmail.it; 6 Department of Translational Medicine and Surgery, Fondazione Policlinico Universitario A. Gemelli IRCCS, Università Cattolica del Sacro Cuore, 00168 Rome, Italy; eleonora.gaetani@unicatt.it; 7 Unit of Internal Medicine, Cristo Re Hospital, 00167 Rome, Italy; 8 Unit of Gynecology and Obstetrics, Ospedale San Giuseppe Moscati, 81031 Aversa, Italy; dr.rosannaesposito@gmail.com; 9 Division of Gynecologic Oncology, Department of Woman and Child Health and Public Health, Fondazione Policlinico Universitario A. Gemelli IRCCS, 00168 Rome, Italy

**Keywords:** autoimmunity, estrogen fluctuations, female brain, microglia activation, mood disorders, neuroimmune signaling, neuroinflammation, sex differences, stress vulnerability, women’s mental health

## Abstract

Women exhibit a higher prevalence of depression, anxiety, stress-related disorders, and autoimmune conditions compared to men, yet the biological mechanisms underlying this sex difference remain incompletely understood. Growing evidence identifies neuroinflammation as a central mediator of psychiatric vulnerability in women, shaped by interactions between sex hormones, immune activation, and neural circuit regulation. Throughout the female lifespan, fluctuations in estrogen and progesterone, such as those occurring during puberty, the menstrual cycle, pregnancy, postpartum, and perimenopause, modulate microglial activity, cytokine release, and neuroimmune signaling. These hormonal transitions create windows of heightened sensitivity in key brain regions involved in affect regulation, including the amygdala, hippocampus, and prefrontal cortex. Parallel variations in systemic inflammation, mitochondrial function, and hypothalamic–pituitary–adrenal (HPA) axis responsivity amplify stress reactivity and autonomic imbalance, contributing to increased risk for mood and anxiety disorders in women. Emerging data also highlight sex-specific interactions between the immune system and monoaminergic neurotransmission, gut–brain pathways, endothelial function, and neuroplasticity. This review synthesizes current neuroscientific evidence on the sex-dependent neuroinflammatory mechanisms that bridge hormonal dynamics, brain function, and psychiatric outcomes in women. We identify critical periods of vulnerability, summarize converging molecular pathways, and discuss novel therapeutic targets including anti-inflammatory strategies, estrogen-modulating treatments, lifestyle interventions, and biomarkers for personalized psychiatry. Understanding neuroinflammation as a sex-specific process offers a transformative perspective for improving diagnosis, prevention, and treatment of psychiatric disorders in women.

## 1. Introduction

Women exhibit a disproportionately higher vulnerability to depression, anxiety, post-traumatic stress disorder, and stress-related somatic syndromes compared to men [1,2]. While psychosocial explanations have historically been emphasized, contemporary neuroscience has shifted attention toward biological pathways that differ between the sexes, with neuroinflammation emerging as a central mechanism linking hormonal fluctuations, immune responsivity, microglial dynamics, and emotional regulation throughout the female lifespan. Previous clinical, neuroimaging, and preclinical studies have consistently reported sex differences in immune responsivity, stress reactivity, and mood disorder prevalence [3,4]. However, these findings have largely been examined in isolation rather than integrated into a unified neuroinflammatory framework.

Sex differences in immune function are evident from early development and persist into adulthood, with women generally displaying stronger innate and adaptive immune responses than men [5]. Although advantageous for pathogen defense, this enhanced responsiveness increases susceptibility to autoimmune disorders and exaggerated inflammatory reactions under stress [6]. In the CNS, these peripheral immune patterns interact with microglia, astrocytes, and endothelial cells to shape neural plasticity, neurotransmission, and ultimately affective behavior [7,8].

Across the female lifespan, estrogen and progesterone fluctuations represent some of the most influential modulators of neuroinflammatory tone. Estradiol can exert both anti-inflammatory and pro-inflammatory effects depending on dose, receptor distribution, and brain-region context [9]. These bidirectional effects help explain why hormonal transitions (puberty, menstrual cycle, pregnancy, postpartum, and perimenopause) represent periods of heightened vulnerability to mood instability [10]. During these transitions, shifts in sex hormones shape microglial reactivity, cytokine release (e.g., IL-6, TNF-α), mitochondrial function, and the organization of limbic–prefrontal circuits critical for emotion regulation [11,12,13].

Stress physiology constitutes another domain of pronounced sex divergence. Women tend to exhibit greater HPA-axis responsivity, particularly to interpersonal stress, which synergizes with inflammatory pathways to amplify mood dysregulation [14]. Chronic stress primes microglia toward pro-inflammatory states that disproportionately affect females, increasing vulnerability to depression-like phenotypes in both humans and animal models [15,16]. These stress–immune interactions intersect with monoaminergic systems, especially serotonin and dopamine, which are themselves modulated by estradiol and play a crucial role in mood regulation [12,17].

Advances in neuroimaging, molecular psychiatry, and systems immunology have begun to unravel the sex-dependent biological architecture underlying these outcomes, including sex-specific patterns of inflammatory signaling, stress responsivity, and emotion-related circuit connectivity, as demonstrated by neuroimaging studies [18], transcriptomic analyses [15,16], and immunological investigations [5,6]. Yet integrated models that connect endocrine fluctuations, immune responsivity, microglial biology, and neural circuit function remain limited. Specifically, unresolved questions concern how hormonal fluctuations interact with microglial priming, HPA-axis regulation, and monoaminergic signaling to shape sex-specific vulnerability across distinct developmental and reproductive windows. This review directly addresses these gaps by integrating endocrine, immune, and circuit-level evidence across the female lifespan.

In particular, the present work aims to synthesize current evidence on the neuroinflammatory mechanisms that uniquely shape the female brain, emphasizing how hormonal transitions, immune activation, metabolism, and neurocircuitry interact to influence vulnerability to mood and anxiety disorders. By mapping converging molecular pathways and identifying windows of heightened sensitivity, this review contributes to supporting sex-informed prevention and treatment strategies, including anti-inflammatory approaches, hormone-modulating therapies, lifestyle interventions, and emerging biomarkers for personalized psychiatry.

## 2. Materials and Methods

This article is a narrative review aimed at synthesizing recent evidence on sex-specific neuroinflammatory mechanisms underlying vulnerability to mood and stress-related disorders in women. Although not following a systematic review design, the methodology adhered to principles of transparency and reproducibility recommended for narrative syntheses in biomedical research.

### 2.1. Search Strategy and Information Sources

A literature search was conducted between January and March 2025 across the following electronic databases: PubMed/MEDLINE, Scopus, Web of Science, and PsycINFO. Additional relevant articles were identified by examining the reference lists of included papers and recent reviews in the field.

Searches combined terms related to neuroinflammation, sex differences, female brain, mood and anxiety disorders, hormonal transitions, and microglial activity. The main search string was: “neuroinflammation” AND (“women” OR “female” OR “sex differences”) AND (“mood disorders” OR “anxiety” OR “stress”) AND (“estrogen” OR “progesterone” OR “microglia”). Equivalent MeSH terms and controlled vocabulary were applied when available.

### 2.2. Eligibility Criteria

To ensure scientific relevance and temporal coherence, the following criteria were applied: for inclusion criteria, peer-reviewed articles published between 2014 and 2025; studies involving humans or mammalian models; research focusing on neuroinflammatory mechanisms, sex differences, hormonal regulation, stress responsivity, or affective disorders; original research (clinical, neuroimaging, genetic, molecular) and high-quality reviews. High-quality reviews were defined as narrative or systematic reviews published in peer-reviewed journals that demonstrated clear methodological transparency, comprehensive literature coverage, explicit consideration of sex-specific biological mechanisms, and integration of molecular, neuroimmune, and neuropsychiatric evidence.

Exclusion criteria were: articles not available in English; studies focusing exclusively on pediatric or geriatric populations without sex-specific analyses; papers centered purely on psychosocial or epidemiological aspects without biological correlates; and conference abstracts, dissertations, commentaries, and non–peer-reviewed material.

### 2.3. Study Selection and Data Extraction

Titles and abstracts were screened for eligibility, followed by full-text evaluation to confirm relevance. Discrepancies were resolved through consensus.

### 2.4. Assessment of Bias and Quality

Although formal risk-of-bias tools were not applied (as recommended for systematic reviews), priority was given to studies with clearly defined sex-stratified analyses, neurobiological findings replicated across independent samples or models, articles published in well-established, peer-reviewed journals with a strong focus on neuroscience, psychiatry, immunology, or neuroendocrinology, characterized by rigorous editorial standards and broad scientific influence within the field. Potential sources of bias (such as heterogeneity in hormonal status, menstrual cycle phase, contraceptive use, and age) were considered when interpreting findings. A limitation of this narrative approach is the absence of formal quantitative synthesis and standardized risk-of-bias assessment. However, this design was chosen to enable an integrative and mechanistic interpretation of heterogeneous evidence that would not be amenable to meta-analytic methods.

## 3. Sex-Specific Neuroimmune Mechanisms

Sex differences in neuroimmune function emerge early in development and persist throughout the lifespan, shaping distinct trajectories of vulnerability to mood and anxiety disorders, as supported by developmental neuroimmunology studies [7,19,20,21].

These differences are not merely hormonal by-products but reflect intrinsic, genetically and epigenetically regulated immune programs that influence how the female brain responds to stress, infection, metabolic signals, and environmental adversity [19,20]. In the CNS, neuroimmune interactions are orchestrated primarily by microglia, astrocytes, endothelial cells, and peripheral immune mediators, all of which display sex-dependent activity patterns [21].

### 3.1. Innate Immune Responsivity and Microglial Reactivity

One of the most robust findings is that females exhibit stronger innate immune responses than males, both peripherally and centrally. Evidence from rodent models indicates that female microglia display heightened immune surveillance, enhanced phagocytic activity, and a more readily inducible pro-inflammatory transcriptional profile following stress or immune challenge. Complementary evidence from human post-mortem, neuroimaging, and peripheral biomarker studies suggests that inflammatory signaling is more strongly associated with affective symptoms and neural circuit alterations in women than in men [22]. Microglial development is also sexually dimorphic. Preclinical animal studies show that males exhibit greater microglial density in early postnatal life, whereas females display greater microglial priming and inflammatory responsivity during adolescence and adulthood, particularly following stress exposure [8].

This baseline hyperresponsivity contributes to a heightened magnitude of microglial activation and inflammatory signaling in response to stress or immune challenge in females, including NF-κB signaling and cytokine cascades (IL-1β, IL-6, TNF-α), which are consistently linked to affective symptoms and altered neural plasticity [3]. Importantly, microglial reactivity in females appears to interact more strongly with endocrine states (e.g., fluctuations in estradiol), resulting in dynamic, hormone-sensitive inflammatory profiles that are not observed in the same form in males [23]. These findings are supported by converging evidence from developmental and adult microglial studies demonstrating sex-dependent immune reactivity and transcriptional profiles [7,8,22].

### 3.2. Adaptive Immunity and Autoimmune Predisposition

Females demonstrate enhanced adaptive immunity, with stronger B-cell and T-cell activation, higher antibody production, and greater vulnerability to autoimmunity [19].

Autoimmune disorders such as lupus, multiple sclerosis, and autoimmune thyroiditis, which are more prevalent in women, are frequently associated with affective disturbances in human clinical populations, including depressive symptoms, anxiety, fatigue, and cognitive impairment. These clinical observations are supported by animal models demonstrating that chronic peripheral inflammation alters limbic and prefrontal circuits involved in emotional regulation [24]. This co-occurrence suggests shared inflammatory pathways that may influence mood symptoms through cytokine signaling, blood–brain barrier permeability, and neurovascular coupling. Although these associations are derived from heterogeneous study designs, converging clinical and experimental evidence consistently links autoimmune and inflammatory activity to affective disturbances, particularly in female populations.

Emerging evidence indicates that inflammatory autoimmune flares are associated with sex-dependent changes in neural circuits that regulate emotion, particularly the amygdala and prefrontal cortex [25]. These findings provide a compelling biological link between autoimmune predisposition and the higher prevalence of internalizing disorders observed in women. Clinically, these immune-mediated mechanisms have been associated with affective disturbances including depressive symptoms, anxiety, fatigue, anhedonia, cognitive slowing, and stress intolerance, frequently observed in women with autoimmune and inflammatory disorders.

### 3.3. Peripheral–Central Immune Crosstalk

Sex differences are evident not only within the CNS but also in the bidirectional communication between peripheral and central immune systems. Females exhibit greater permeability of inflammatory signals across the blood–brain barrier (BBB) during periods of hormonal transition, which may facilitate stronger CNS immune activation [26]. Moreover, estrogen can modulate endothelial tight junctions and microglial–vascular interactions, influencing the propagation of peripheral cytokines into brain tissue [27].

This enhanced immune crosstalk contributes to stronger sickness behavior responses, heightened affective sensitivity to infection, and greater likelihood that peripheral inflammation—such as obesity-related low-grade inflammation or postpartum immune shifts—will influence mood and cognition in women [28].

### 3.4. Epigenetic and Genetic Contributors to Sex Differences

Recent genomic and epigenomic studies, primarily derived from human brain tissue and peripheral immune cells, have demonstrated that sex chromosomes contribute to neuroimmune dimorphism. Complementary animal models further show that X-linked immune genes escaping inactivation enhance inflammatory responsivity and stress sensitivity in females [29]. Additionally, inflammatory genes such as TLR7 and CXCL13, which lie on the X chromosome and escape inactivation, show higher expression in females and may confer increased susceptibility to inflammation-related mood disorders [30].

Epigenetic modifications, particularly DNA methylation and histone acetylation in stress- and immune-related genes, also differ by sex and respond to hormonal states across the lifespan [31]. These mechanisms help explain why early-life adversity shows stronger long-term neuroinflammatory consequences in females, contributing to persistent HPA-axis dysregulation, microglial priming, and altered limbic circuitry.

### 3.5. Neuroimmune Modulation of Emotional Processing

Collectively, sex-dependent immune mechanisms converge to shape emotional processing. Inflammatory activation alters amygdala responsivity, reduces hippocampal neurogenesis, and disrupts prefrontal regulatory control, all processes that show greater sensitivity to cytokines in females [32]. This immune–circuit interplay contributes to the female-typical pattern of internalizing symptoms, such as rumination, anhedonia, and anxiety, particularly during periods of hormonal flux [33].

Ultimately, sex-specific neuroimmune dynamics do not simply elevate risk; they define a distinct biological trajectory of emotional regulation in females, modulating vulnerability and resilience throughout life.

## 4. Hormonal Transitions and Neuroinflammatory Windows

Hormonal fluctuations across the female lifespan represent one of the most powerful modulators of neuroimmune dynamics, creating periods in which inflammatory signaling, neural plasticity, and emotional regulation become particularly sensitive to perturbation. These transitions, including puberty, the menstrual cycle, pregnancy, postpartum, and perimenopause, interact with microglial function, peripheral cytokine activity, and limbic–prefrontal circuitry, shaping sex-specific vulnerability to mood and anxiety disorders [9,10].

### 4.1. Puberty: The First Major Neuroinflammatory Shift

Puberty marks the first major surge in estradiol, reorganizing neural circuits involved in stress responsivity and emotional regulation. Estradiol exerts complex effects on microglia, promoting anti-inflammatory or pro-inflammatory phenotypes depending on receptor distribution, local neural environment, and timing within developmental windows [9].

Females exhibit greater microglial priming in response to pubertal stressors, with heightened sensitivity to cytokine release and long-term alterations in affective behavior [8]. These effects are consistent with evidence that early-life inflammatory reactivity disproportionately influences emotional outcomes in girls [19,34].

### 4.2. Menstrual Cycle: Recurrent Immune–Hormonal Oscillations

The menstrual cycle introduces recurrent, rapid hormonal fluctuations that exert measurable effects on immune function. Estradiol and progesterone regulate cytokine production, T-cell activation, and microglial responsiveness [23].

During the late luteal phase, when both estradiol and progesterone decline sharply, human studies report increases in inflammatory markers such as IL-6 and TNF-α, alongside reduced prefrontal regulatory activity and heightened limbic reactivity. These findings parallel mechanistic evidence from animal models showing hormone-dependent modulation of microglial activation and cytokine signaling [12]. These neuroimmune oscillations can exacerbate negative affect and stress sensitivity, particularly in women with pre-existing inflammatory vulnerability [5].

For a subset of women, these physiological processes manifest clinically as premenstrual mood symptoms and reduced stress tolerance [10].

### 4.3. Pregnancy: Immune Recalibration and Neuroprotection

Pregnancy induces profound alterations in immune signaling, characterized by alternating anti-inflammatory and pro-inflammatory phases coordinated by estradiol and progesterone. Mid-pregnancy is generally associated with anti-inflammatory bias, involving reduced microglial activation and dampened pro-inflammatory responses [27].

However, early and late gestation are marked by transient pro-inflammatory shifts, necessary for implantation and parturition, respectively. These fluctuations influence blood–brain barrier permeability, metabolic stress, and neural immune surveillance [27,35].

Although pregnancy often confers temporary protection against mood disorders, dysregulated immune transitions can precipitate affective symptoms, particularly in women with pre-existing inflammatory sensitivity [4].

### 4.4. Postpartum: A Critical Window of Neuroinflammatory Sensitivity

The postpartum period is characterized by one of the most abrupt endocrine transitions in human physiology. Estradiol and progesterone levels plummet within hours after childbirth, while immune activity rebounds toward a pro-inflammatory state [10].

Evidence indicates increased microglial activation, elevated cytokine release, and disrupted functional connectivity between the amygdala and prefrontal cortex during this period [11].

Women with heightened baseline immune responsivity or stress-induced microglial priming show marked susceptibility to postpartum mood disorders [15,16].

Neuroimaging research demonstrates enhanced amygdala reactivity to emotional stimuli postpartum, consistent with inflammation-driven modulation of limbic circuits [33].

### 4.5. Perimenopause: Chronic Low-Grade Inflammation and Neural Plasticity Decline

Perimenopause introduces fluctuating and eventually declining estrogen levels, which contribute to increased basal inflammation, mitochondrial stress, and reduced synaptic plasticity in mood-relevant brain regions [12,13].

Estradiol withdrawal enhances microglial reactivity and increases vulnerability to stress-induced inflammatory signaling, amplifying the risk of depression during the menopausal transition [14]. This period is characterized by disruptions in serotonin and dopamine pathways [12,17], diminished trophic support in the hippocampus, and altered HPA-axis feedback regulation, contributing to a second major peak in female depressive vulnerability described across epidemiological studies [1,2].

In later life, particularly during the menopausal transition, preventive strategies targeting inflammatory burden, stress regulation, and lifestyle factors may play a crucial role in reducing long-term vulnerability to mood disorders.

**Figure 1:**
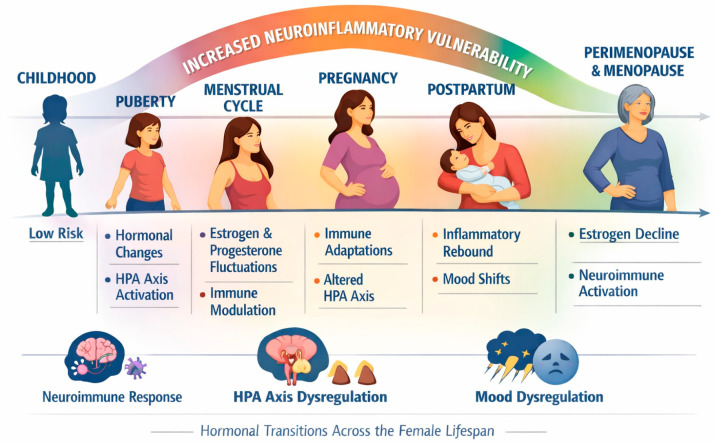
Windows of Neuroinflammatory Vulnerability Across the Female Lifespan. Note. Hormonal transitions, including puberty, menstrual cycle oscillations, pregnancy-related immune shifts, the postpartum inflammatory rebound, and perimenopausal estrogen decline, create temporally sensitive periods during which neuroimmune, stress-response, and mood-regulating systems display heightened reactivity. Abbreviations: HPA axis, hypothalamic–pituitary–adrenal axis.

As shown in Figure 1, puberty, the menstrual cycle, pregnancy, the postpartum period, and perimenopause each represent distinct temporal windows during which neuroimmune activity becomes particularly reactive.

## 5. Microglia, HPA Axis, and Neurotransmission in the Female Brain

Sex differences in neuroinflammatory vulnerability arise not only from hormonal fluctuations and immune responsivity but also from the intersection of microglial activity, stress-system regulation, and neurotransmitter dynamics. Together, these systems form an integrated biological framework that explains why women exhibit heightened sensitivity to stress and greater risk for mood and anxiety disorders across the lifespan [3,14].

### 5.1. Microglial Function and Sex-Specific Immune Signaling

Microglia serve as the principal immune sentinels of the brain and display pronounced sex differences in density, morphology, transcriptional activity, and reactivity to stressors. Female microglia tend to exhibit enhanced immune surveillance and faster transition to activated phenotypes in response to cytokines, psychosocial stress, and hormonal withdrawal [8,22].

Estradiol modulates microglial function in a bidirectional manner, exerting anti-inflammatory effects under steady-state conditions but potentiating inflammatory responsiveness during hormonal transitions such as puberty, the menstrual cycle, postpartum, and perimenopause [9,10,27].

Research indicates that microglial activation in females more readily affects synaptic pruning, glutamatergic transmission, and dendritic spine remodeling, particularly in limbic regions that regulate affective processing [23,25]. This microglia–synapse coupling may be central to sex-specific pathways of emotional vulnerability, influencing how stress shapes neural circuitry over time [29,36].

### 5.2. HPA Axis Reactivity: A Sex-Divergent Stress Pathway

The HPA axis orchestrates physiological responses to stress and exhibits robust sex differences in responsivity and feedback regulation. Women show greater cortisol reactivity, slower recovery, and enhanced sensitivity to interpersonal stressors, reflecting interactions between estradiol, glucocorticoid receptors, and neuroimmune signaling [14].

Estradiol increases CRH gene expression and impairs glucocorticoid receptor negative feedback, thereby amplifying stress responses and creating conditions under which inflammatory cascades more readily influence mood circuits [10,12].

Chronic HPA activation primes microglia toward pro-inflammatory states, particularly in females, where estradiol withdrawal or fluctuation increases cytokine-driven modulation of neural plasticity [15,16].

Moreover, early-life stress produces sex-specific epigenetic signatures on HPA-related genes, including NR3C1 (glucocorticoid receptor) and FKBP5, which regulate stress responsivity across development [31,37]. These molecular imprints contribute to the enduring nature of stress sensitivity in women and its interaction with neuroinflammation.

### 5.3. Serotonin, Dopamine, and Monoaminergic–Immune Crosstalk

Monoaminergic systems, particularly serotonin (5-HT) and dopamine (DA), play key roles in mood regulation and are profoundly influenced by estradiol and inflammatory states. Estradiol enhances tryptophan hydroxylase activity, increases 5-HT synthesis, and modulates serotonergic receptor expression, while also regulating dopaminergic tone in prefrontal and mesolimbic pathways [12,17].

Inflammation disrupts these systems via multiple mechanisms. Animal models demonstrate cytokine-induced activation of indoleamine-2,3-dioxygenase (IDO), reduced serotonin availability, and altered dopaminergic signaling, while human PET and neuroimaging studies reveal stronger associations between inflammatory markers, reduced reward sensitivity, and affective symptoms in women [3,32].

**Figure 2:**
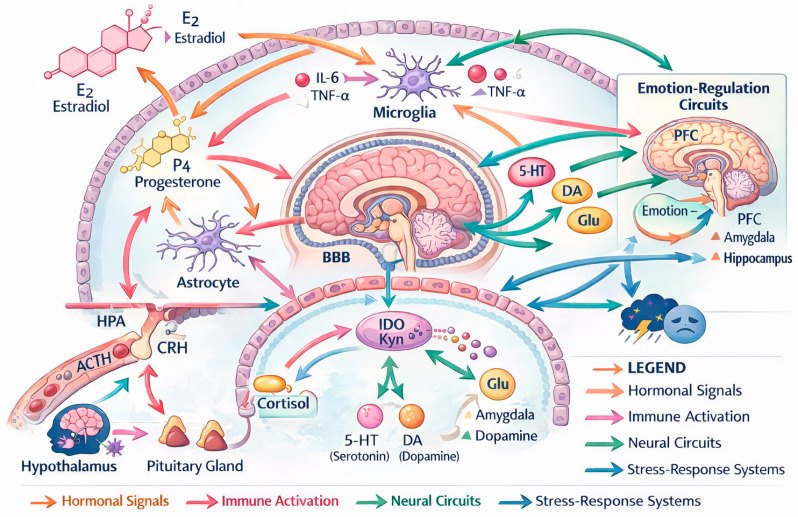
Integrated Neurobiological Network Model. Note. Integrated neurobiological network model illustrating interactions among hormonal signals, microglial reactivity, HPA-axis regulation, monoaminergic neurotransmission, and emotion-regulation circuits. Bidirectional pathways reflect dynamic neuroimmune–neuroendocrine feedback loops contributing to sex-specific vulnerability to mood and stress-related disorders. Hormonal signals also exert direct effects on astrocytes and neurons, modulating synaptic plasticity, metabolic support, glutamate homeostasis, and neurotrophic signaling, thereby contributing to sex-specific regulation of emotion-related neural circuits. Arrows indicate bidirectional regulatory interactions, reflecting dynamic feedback loops among hormonal signals, immune activation, neural circuits, and stress-response systems. Abbreviations: E2, estradiol; P4, progesterone; CRH, corticotropin-releasing hormone; HPA axis, hypothalamic–pituitary–adrenal axis; GR, glucocorticoid receptor; 5-HT, serotonin (5-hydroxytryptamine); DA, dopamine; Glu, glutamate; PFC, prefrontal cortex; BBB, blood–brain barrier; IL-6, interleukin-6; TNF-α, tumor necrosis factor-alpha; IDO, indoleamine 2,3-dioxygenase.

Women appear particularly sensitive to these neurotransmitter–immune interactions. Neuroimaging studies show stronger associations between inflammation and negative affect, reduced reward sensitivity, and dysregulated emotional processing in females relative to males [32,33]. These vulnerabilities are further amplified during hormonal transitions, which dynamically modulate both monoaminergic systems and immune signaling [12,12]. These interactions are summarized schematically in Figure 2.

### 5.4. Limbic–Prefrontal Circuit Vulnerability

Sex-specific inflammatory and hormonal dynamics converge on limbic–prefrontal networks that govern emotion regulation. The amygdala, central to threat detection, shows heightened activation in women during inflammatory challenges and postpartum hormonal transitions [33,38].

The prefrontal cortex (PFC), responsible for top-down regulation, exhibits reduced functional connectivity and decreased inhibitory control under inflammatory load, with these effects disproportionately expressed in females [12,32].

Chronic neuroinflammation disrupts hippocampal neurogenesis, synaptic plasticity, and stress resilience, contributing to rumination, anxiety, and cognitive vulnerability [11,25]. These circuit-level changes reflect a cumulative interaction between microglial activity, hormonal flux, and dysregulated neurotransmission.

### 5.5. An Integrative Model of Female Neurobiological Vulnerability

Across the female lifespan, hormonal fluctuations, immune responsivity, and stress physiology do not act as isolated processes. Instead, they converge in a dynamic neurobiological landscape that shapes how women experience stress, regulate emotions, and recover, or fail to recover, from adversity. This interplay can be conceptualized not as a simple cascade, but as a multidimensional dialog among microglia, the HPA axis, and monoaminergic systems, each reciprocally influencing the others across development and hormonal transitions.

A central feature of this model is the heightened sensitivity of female microglia to internal and external perturbations. Microglia in females respond more vigorously to cytokines, metabolic stressors, and hormonal withdrawal, adopting activated phenotypes that directly influence synaptic remodeling, glutamate balance, and neuroplasticity [8,22]. Recent transcriptomic studies indicate that female microglia maintain a “primed” molecular state, poised to shift into inflammatory profiles more readily than in males, even in the absence of overt stressors [36,39]. This priming may underlie why emotional stress and immune activation have stronger effects on limbic circuits in women.

Parallel to microglial sensitivity, the HPA axis operates at a higher baseline gain in females, producing stronger and more sustained cortisol responses to interpersonal or emotional stressors [14]. Estradiol amplifies CRH expression, alters glucocorticoid receptor function, and increases central feedback resistance, thereby magnifying the neurobiological imprint of stressors [10,37]. Chronic HPA activation feeds back on microglia, reinforcing pro-inflammatory states and lowering the threshold for future stress reactivity. This cyclical interaction—as much hormonal as immunological—creates a biological context in which stress and inflammation continually reinforce one another.

Monoaminergic systems serve as the third major node in this integrated model. Serotonin and dopamine pathways, central to mood regulation and reward processing, are both modulated by estradiol and vulnerable to inflammatory disruption [12,17]. Inflammation decreases serotonin availability through accelerated tryptophan metabolism, reduces dopamine release in reward circuits, and enhances glutamate excitotoxicity—all pathways shown to produce stronger affective consequences in females [32,40]. Neuroimaging studies increasingly reveal that women exhibit greater functional coupling between inflammatory markers and limbic hyperreactivity, particularly in the amygdala and ventromedial prefrontal cortex [28,33]. These regions are crucial for emotional salience detection and regulatory control, meaning that neuroinflammation does not simply alter mood, but recalibrates the neural architecture of emotional processing.

When these systems are viewed together, a clearer picture emerges: microglia respond more robustly to stress and hormonal withdrawal; the HPA axis amplifies inflammatory responses and sustains arousal states; monoaminergic pathways are more vulnerable to immune-mediated disruption; limbic–prefrontal circuits integrate these biological signals into subjective emotional experience.

This integrated model helps explain why periods of hormonal transition (puberty, perimenopause, postpartum) coincide with peaks in female emotional vulnerability. These are phases in which microglial priming, HPA dysregulation, and neurotransmitter shifts align synergistically, rendering the female brain more reactive to both biological and psychosocial stressors. Conversely, it also suggests that interventions targeting inflammation, HPA modulation, or estrogen signaling may have outsized therapeutic benefits in women, particularly when timed to these transitions.

Ultimately, this framework supports a shift toward sex-informed neuropsychiatry, where emotional vulnerability in women is understood not as a consequence of isolated risk factors but as a complex, interconnected biological system shaped by hormonal rhythms, immune landscapes, and neural circuitry dynamics [25]. Far from representing pathology, these mechanisms reflect evolutionary trade-offs—enhanced immunity, heightened social attunement, reproductive adaptation—that confer both strengths and vulnerabilities across the lifespan. Figure 2 illustrates how hormonal fluctuations, microglial activation, HPA-axis responsivity, and neurotransmitter dynamics converge within emotion-regulation circuits, forming an interconnected network that shapes women’s neuroinflammatory vulnerability.

## 6. Discussion

### 6.1. Neuroimmune Priming and Sex Differences

Understanding why women are disproportionately affected by depression, anxiety, and stress-related disorders requires integrating biological, hormonal, and immunological perspectives into a coherent neuropsychiatric framework. The evidence reviewed across sections highlights that neuroinflammation operates as a central hub, shaping how the female brain responds to both internal and external stressors. While these mechanisms have been described individually in prior literature, recent research suggests that their synergistic interaction, rather than their isolated effects, is what truly differentiates female vulnerability trajectories.

### 6.2. Hormonal Transitions and Vulnerability Windows

A central observation emerging from contemporary studies is that female microglia remain in a more reactive or “sensitized” state across the lifespan. This state is reflected in transcriptomic, epigenetic, and morphological signatures pointing toward heightened responsiveness to hormonal shifts and inflammatory mediators [36,39]. Newer work extends this notion by showing that microglial priming can be detected even in healthy women during natural hormonal transitions such as the menstrual cycle or perimenopause, a phenomenon not equivalently observed in men [41]. Such findings underscore the idea that sex differences in neuroinflammation are not merely reactive but are baseline features of female neurobiology.

### 6.3. Stress–Immune Interactions

Parallel research on the HPA axis has revealed a similarly robust sex-specific pattern. Beyond demonstrating increased cortisol responsivity in women, recent experimental paradigms incorporating social evaluation or interpersonal stress show that females exhibit prolonged neural and endocrine recovery times, with persistent activation of immune-related transcriptional programs [42]. These prolonged responses appear particularly pronounced during periods of hormonal instability and are amplified by early-life adversity, which leaves sex-specific epigenetic marks on genes regulating HPA feedback sensitivity [37,43]. The combined impact of microglial priming and HPA dysregulation creates a biological context in which stress and inflammation potentiate each other, leading to sustained affective dysregulation.

### 6.4. Neurotransmitter and Circuit-Level Modulation

Monoaminergic systems add an additional layer of complexity. While sex differences in serotonin and dopamine signaling are well established [12,17], recent advances in molecular imaging indicate that inflammation affects serotonin transporter availability, dopamine synthesis capacity, and glutamatergic tone in ways that differ markedly between sexes [28,40]. For example, a large PET study demonstrated that inflammatory challenges reduced striatal dopamine synthesis more profoundly in women, correlating with reduced reward sensitivity and increased anhedonia [44]. These findings not only support the proposed integrated model but also highlight potential targets for sex-informed treatments, such as anti-inflammatory strategies to restore reward-related signaling.

Another important dimension emerging from recent research is the role of brain network connectivity. Functional MRI studies increasingly show that women display stronger coupling between inflammatory markers and amygdala–prefrontal network dysregulation, particularly during hormonally dynamic periods such as postpartum or perimenopause [28,33]. New longitudinal evidence suggests that even subtle variations in inflammatory tone predict changes in default mode network coherence and emotion-regulation circuitry over time, but with significantly greater effects in women [45]. Such findings bridge molecular-level mechanisms with macroscale neural dynamics and help explain why subjective emotional vulnerability often emerges during biologically predictable transitions.

### 6.5. Translational and Clinical Implications

Despite this progress, gaps remain. Most mechanistic research has been conducted in male animals or in mixed-sex samples without sex-stratified analyses. Only in the past decade have rigorous models begun incorporating sex as a biological variable, and even fewer studies consider dynamic hormonal states or the intersection between inflammation and psychosocial context. Emerging work on the gut–brain axis suggests additional sex-specific interactions: women may have stronger inflammatory responses to microbiome perturbations and greater affective consequences following gut dysbiosis [46]. This opens new avenues for personalized interventions targeting diet, microbiota, or peripheral inflammation.

From a therapeutic perspective, this integrative framework suggests that sex-specific windows of vulnerability (puberty, postpartum, and perimenopause) are also windows of opportunity. Anti-inflammatory treatments, neuroimmune modulators, estradiol-based interventions, and HPA-targeting strategies may have greater efficacy when delivered during these transitions. Importantly, recent randomized trials indicate that anti-inflammatory agents such as minocycline or omega-3 fatty acids may produce stronger antidepressant effects in women than in men, potentially due to differential modulation of microglial activity [47,48]. Similarly, neurosteroids such as allopregnanolone show profound postpartum mood benefits, illustrating the clinical relevance of understanding hormonal–neuroimmune interactions [49].

Taken together, the emerging data support a shift toward sex-informed biological psychiatry, in which emotional vulnerability is conceptualized as the outcome of interactions among hormones, immune dynamics, neural circuits, and environmental stressors. Far from representing a pathological deficiency, this heightened responsivity may reflect evolutionary advantages in social sensitivity, immune defense, and reproductive adaptation, but at the cost of increased risk for affective dysregulation in a modern stress-laden environment. By fully embracing the complexity of these interactions, future research and clinical practice can move toward genuinely personalized approaches that recognize and treat the unique neurobiological needs of women. These findings underscore the importance of considering intersectional biological and psychosocial factors, including age, hormonal status, metabolic health, stress exposure, and social context, rather than relying on a single explanatory model in either research or clinical settings.

A summary of the principal sex-specific neuroinflammatory mechanisms discussed in this review is presented in Table 1. The table integrates microglial signaling, hormonal transitions, HPA-axis responsivity, monoaminergic modulation, and circuit-level alterations across the female lifespan.

## 7. Conclusions

This review integrates current evidence to highlight neuroinflammation as a central, sex-informed mechanism linking hormonal dynamics, immune responsivity, stress regulation, and neural circuit function across the female lifespan.

The evidence presented in this review converges on a compelling principle: neuroinflammation acts as a central integrator of sex differences in emotional vulnerability, linking hormonal fluctuations, immune responsivity, neurotransmission, and neural circuit dynamics across the female lifespan. The novelty of this review lies in its integrative framework, which synthesizes neuroimmune, endocrine, and neural circuit evidence across distinct hormonal transitions, offering a lifespan-based and sex-informed perspective not previously consolidated in a single model.

Rather than being explained by a single mechanism, women’s heightened susceptibility to depression, anxiety, and stress-related disorders emerges from the intersection of multiple biological processes, each amplified during specific hormonal transitions.

What becomes increasingly clear is that female emotional vulnerability is dynamic rather than static. Puberty, the menstrual cycle, pregnancy, postpartum, and perimenopause all introduce shifts in estradiol and progesterone that modulate microglial activation, cytokine signaling, stress responsivity, and monoaminergic tone [9,10,12]. These transitions create windows during which the brain becomes more sensitive to internal and external stressors. New longitudinal studies demonstrate that even mild inflammatory fluctuations can predict changes in emotional reactivity and neural network organization in women but not in men, highlighting the unique temporal patterns of female neurobiology [16,45].

The expanding field of sex-informed neuropsychiatry shows that neuroinflammation in women is not merely a pathological vulnerability; it may also reflect evolutionary adaptations, including enhanced pathogen defense, greater sensitivity to social cues, and reproductive demands that rely on flexible neuroimmune communication [25,46]. Yet in contemporary environments characterized by chronic stress, sleep disruption, metabolic inflammation, and psychosocial overload, these adaptive mechanisms can tilt toward maladaptive trajectories, contributing to the rising global burden of mood disorders in women [50].

From a clinical perspective, these insights underscore the urgent need for sex-specific prevention and treatment approaches. Anti-inflammatory pharmacotherapies, neurosteroid treatments, dietary and lifestyle interventions targeting immune pathways, and HPA-axis modulators all hold particular promise for women, especially when applied during hormonally sensitive periods [47,48,49]. Moreover, recognizing the influence of menstrual cycle phase, postpartum neurobiology, or perimenopausal transition may enhance the precision of antidepressant selection and improve therapeutic outcomes [51].

Ongoing research must move beyond treating sex as a demographic variable and instead approach it as a core biological construct. This requires more studies with stratified analyses, hormonal-state tracking, multimodal biomarker integration, and longitudinal designs that capture within-woman variability across time. Promising areas of expansion include sex-specific neuroimmune imaging, microbiome–immune interactions, and neurosteroid modulation of microglial activity, all of which may yield transformative insights into women’s mental health [52,53].

In conclusion, neuroinflammation provides a powerful framework for understanding the biological foundations of emotional vulnerability in women. By integrating hormonal, immune, neurotransmitter, and neural circuit perspectives, we are closer to developing therapeutic strategies that truly reflect the complexity and specificity of the female brain. Such an approach not only advances neuroscience but also moves us toward more equitable and effective mental healthcare for women across the lifespan. Future research adopting longitudinal, sex-informed, and mechanistically driven approaches will be essential to translate these insights into more precise preventive and therapeutic strategies for mood and stress-related disorders.

## 8. Limitations

This review has limitations inherent to its narrative design. The absence of a formal systematic methodology and quantitative meta-analysis precludes statistical estimation of effect sizes or direct comparison across studies. No standardized risk-of-bias assessment was performed, which may influence the relative weight of individual findings. Heterogeneity in study design, species (human versus animal models), hormonal status, and sex-stratified reporting limits the generalizability of some interpretations. Finally, the rapidly evolving nature of neuroimmunology and sex-informed neuroscience means that emerging evidence may not yet be fully represented. Despite these limitations, the narrative approach allowed for an integrative, mechanism-oriented synthesis of complex and heterogeneous data.

## Figures and Tables

**Table 1:** Sex-specific neuroinflammatory mechanisms underlying emotional vulnerability: evidence from human and animal studies.

Mechanism	Description	Key Evidence	Evidence Source	References
Microglial priming and heightened reactivity	Female microglia exhibit greater baseline immune surveillance and enhanced inflammatory responsiveness to stress, hormonal withdrawal, or immune challenge.	Sex-specific microglial transcriptional profiles; enhanced cytokine signaling; increased synaptic pruning in emotion-related circuits.	Animal models; human post-mortem and neuroimaging studies	[8,22,36,39]
Estradiol-dependent immune modulation	Estradiol exerts bidirectional effects on neuroinflammation depending on dose, receptor subtype, brain region, and hormonal state.	Hormonal transitions dynamically alter microglial activation and cytokine production.	Animal models; human endocrine and biomarker studies	[9,10,12,23,27]
Greater HPA-axis responsivity	Females show stronger and more prolonged stress-induced cortisol responses, particularly during hormonally sensitive periods.	Enhanced CRH expression; impaired glucocorticoid receptor feedback; stress-induced inflammatory transcription.	Human endocrine, imaging, and epigenetic studies	[14,37,42,43]
Inflammation–monoamine interactions	Cytokines reduce serotonin synthesis, impair dopamine signaling, and alter glutamatergic tone, with stronger affective consequences in females.	Reduced DA synthesis under inflammatory challenge; IDO activation; altered reward circuitry.	Animal models; human PET and neuroimaging studies	[12,17,40,44]
Limbic–prefrontal circuit sensitivity	Inflammatory signaling preferentially disrupts amygdala–prefrontal connectivity and regulatory control in females.	Heightened amygdala reactivity; reduced PFC inhibitory regulation under inflammatory load.	Human fMRI and biomarker studies	[12,28,32,33,45]
Neuroinflammatory windows across the lifespan	Hormonal transitions create temporally sensitive periods of amplified neuroimmune reactivity.	Pubertal immune shifts; menstrual cytokine oscillations; postpartum inflammatory rebound; perimenopausal inflammation.	Human clinical and biomarker studies; animal models	[10,11,27,34,38]
Epigenetic and chromosomal contributors	X-linked immune genes and sex-specific epigenetic regulation increase inflammatory responsivity.	Escape from X-inactivation (e.g., TLR7); sex-specific methylation of stress-related genes.	Human genetic and epigenetic studies; animal models	[29,30,31,37]
Gut–brain–immune sex differences	Sex-dependent microbiome–immune interactions influence neuroinflammation and affective outcomes.	Differential microbiome composition; stronger inflammatory-affective coupling in females.	Human clinical studies; animal models	[46]
Clinical consequences	Higher prevalence of mood and anxiety disorders and differential treatment responses associated with inflammatory burden.	Enhanced antidepressant response to anti-inflammatory agents; efficacy of neurosteroid therapies postpartum.	Human clinical trials and meta-analyses	[1,2,47,48,49]

Abbreviations: CRH, corticotropin-releasing hormone; DA, dopamine; DMN, default mode network; GR, glucocorticoid receptor; HPA axis, hypothalamic–pituitary–adrenal axis; IDO, indoleamine 2,3-dioxygenase; IL-6, interleukin-6; PFC, prefrontal cortex; PET, positron emission tomography; TNF-α, tumor necrosis factor-alpha; TLR7, Toll-like receptor 7; 5-HT, serotonin (5-hydroxytryptamine).

## Data Availability

No new data were created.
